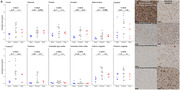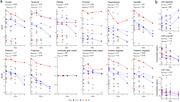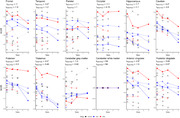# Anti‐amyloid‐β treatment effects on dominantly inherited Alzheimer disease neuropathology: comparing autopsy findings with biomarker outcomes from the DIAN‐TU‐001 trial of gantenerumab or solanezumab

**DOI:** 10.1002/alz.095436

**Published:** 2025-01-09

**Authors:** Charles D. Chen, Erin E. Franklin, Yan Li, Nelly Joseph‐Mathurin, Aime Burns, Diana A Hobbs, Austin A. McCullough, Stephanie A. Schultz, Guoqiao Wang, Tammie L.S. Benzinger, Randall J. Bateman, Richard J. Perrin

**Affiliations:** ^1^ Washington University in St. Louis, St. Louis, MO USA; ^2^ Washington University in St Louis, St Louis, MO USA; ^3^ Massachusetts General Hospital, Brigham and Women’s Hospital, Harvard Medical School, Boston, MA USA

## Abstract

**Background:**

Clinical trials of anti‐Aβ monoclonal antibodies in Alzheimer disease (AD) typically infer target engagement from Aβ positron emission tomography (PET) and/or cerebrospinal fluid (CSF) Aβ42/40 biomarker outcomes that measure Aβ deposits indirectly and/or incompletely. Assessment of postmortem tissue is needed to directly investigate treatment effects on Aβ deposits.

**Method:**

From a clinical trial of anti‐Aβ monoclonal antibodies in dominantly inherited AD (DIAD), we measured Aβ immunohistochemistry area fractions (10D5 antibody) in 10 brain regions from 10 trial cases (representing gantenerumab (n = 4), solanezumab (n = 4), and placebo/no‐treatment (n = 2) arms) and 10 observational study cases. Gantenerumab, solanezumab, and control (placebo/no‐treatment/observational study) groups were compared on the basis of these Aβ area fractions, as well as their antemortem Aβ PET (^11^C‐PiB), and antemortem CSF (Aβ42/40, p‐tau181, and t‐tau) biomarkers. Five participants in the control group did not undergo PET imaging or CSF collection.

**Result:**

Frontal, temporal, parietal, occipital, hippocampus, caudate, putamen, thalamus, cerebellar gray matter, and anterior cingulate Aβ area fractions were significantly lower in the gantenerumab arm versus control group. Temporal, caudate, putamen, and thalamus Aβ PET standardized uptake value ratios (SUVRs) showed significant decreases in the gantenerumab arm versus controls. When using cerebellar white matter as the reference region instead of cerebellar gray matter, anterior cingulate SUVR also showed significant decrease in the gantenerumab arm versus controls, and effect sizes were larger across all regions. CSF Aβ42/40 showed significant increase in the gantenerumab arm versus controls, CSF p‐tau181 showed no significant differences between groups, and CSF t‐tau showed significant decreases in the gantenerumab arm versus controls.

**Conclusion:**

Neuropathological assessments, consistent with Aβ PET and CSF Aβ42/40 biomarkers, found that gantenerumab treatment in these participants reduced, but did not completely eliminate, Aβ deposits. Neuropathology also uniquely revealed lower levels of cerebellar gray matter Aβ in the gantenerumab group versus controls; this finding suggests that using cerebellar white matter as a reference region may improve the accuracy of Aβ PET in DIAD clinical trials. Both findings illustrate the value of neuropathological assessments to AD clinical trials. Future studies of these cases may provide insights into the downstream effects of antibody‐mediated Aβ removal in DIAD.